# Thresholds of acidification impacts on macroinvertebrates adapted to seasonally acidified tropical streams: potential responses to extreme drought-driven pH declines

**DOI:** 10.7717/peerj.11955

**Published:** 2021-11-23

**Authors:** Carissa Ganong, Minor Hidalgo Oconitrillo, Catherine Pringle

**Affiliations:** 1 Department of Biology, Missouri Western State University, St. Joseph, MO, USA; 2 La Selva Biological Station, Organization for Tropical Studies, Puerto Viejo de Sarapiqui, Heredia, Costa Rica; 3 Odum School of Ecology, University of Georgia, Athens, GA, United States of America

**Keywords:** Macroinvertebrate, Stream, Acidification, Tropical, Climate change

## Abstract

**Background:**

Drought-driven acidification events of increasing frequency and severity are expected as a consequence of climate change, and these events may expose macroinvertebrate taxa to increased acidification beyond their tolerance levels. Recent work in lowland Costa Rica has shown that poorly-buffered tropical streams exhibit natural seasonal variation in pH, with extremely low levels (<4.5) after extreme dry seasons). Our goal was to determine the threshold of pH effects on survival of three tropical stream macroinvertebrate taxa.

**Methods:**

We conducted laboratory mesocosm experiments to determine acidification effects (using diluted HCl) on three focal macroinvertebrate taxa collected from a poorly-buffered stream at La Selva Biological Station: (1) mayfly naiads (Ephemeroptera: Leptophlebiidae: *Traverella holzenthali*), (2) adult shrimp (Decapoda: Palaemonidae: *Macrobrachium olfersii*), and (3) larval midges (Diptera: Chironomidae). We also compared the effect of pH on survival and growth rates of larval midges from a poorly-buffered (pH 4.3–6.9) vs. a naturally well-buffered (pH 5.1–6.9) stream.

**Results/Discussion:**

Mayfly and shrimp survival decreased between pH 4.0 and 3.5, overlapping with the range of lowest pH levels (3.6–4.0) recorded during a previous extreme El Niño Southern Oscillation event in 1998 and suggesting that increasingly extreme acidification events induced by climate change may negatively affect their survival. In contrast, survival of larval midges was unaffected by pH regimes at/above 3.5, indicating tolerance to pH levels experienced in poorly-buffered stream during seasonal acidification, which has presumably occurred over millennia. These findings highlight the potential importance of historical pH regimes in structuring macroinvertebrate communities. These results are relevant not only to lowland Neotropical streams, but also signal the need for further research in lotic ecosystems worldwide where drought-driven pH declines have been documented or are probable in the future.

## Introduction

Anthropogenic impacts on aquatic ecosystems include acidification caused by acid rain (Schindler, 1988) and acid mine drainage (MacCausland & McTammany, 2007). Acidification of freshwater systems is also caused by other mechanisms, including CO_2_ inputs (*e.g.*, Hasler et al., 2018) and dilution of alkalinity by seasonal snowmelt (Lepori, Barbieri & Ormerod, 2003). Acidification can alter many ecosystem characteristics, including food web structure (Gray et al., 2016), decomposition rates (Dangles et al., 2004a), nutrient cycling (Ely, Von Schiller & Valett, 2010), algal biomass (Smucker & Vis, 2011), and biodiversity of fish Baker et al. (1996) and macroinvertebrates (Rosemond et al., 1992; Lepori, Barbieri & Ormerod, 2003; Baldigo et al., 2009). Macroinvertebrates in particular are widely known as indicators of water quality and ecosystem health, and the effects of acidification on benthic macroinvertebrates are well documented. Low pH can lead to shifts in community composition (Rosemond et al., 1992; Kullberg, 2002; Woodward, Jones & Hildrew, 2002), increased drift (Hall et al., 1980; Courtney & Clements, 1998), and reduced survival and emergence (Bell, 1971; Courtney & Clements, 1998; Thomsen & Friberg, 2002). Macroinvertebrates are frequently negatively affected in environments that have been exposed to anthropogenic acidification *via* acid deposition or acid mine drainage (Gray, 1998; Kowalik & Ormerod, 2006), and CO_2_-induced acidification can also cause physiological stress and increased mortality in molluscs (reviewed in Hasler et al., 2018) and decreased feeding responses in crayfish (Allison, Dunham & Harvey, 1992).

However, macroinvertebrate assemblages are often tolerant to acidification in habitats that exhibit historically low or variable pH, such as temporary ponds (Batzer, Palik & Buech, 2004), streams with high concentrations of organic acids (Winterbourn & Collier, 1987; Collier et al., 1990; Dangles, Malmqvist & Laudon, 2004b), and mountain streams acidified by seasonal snowmelt (Petrin, Laudon & Malmqvist, 2007). In these cases, macroinvertebrates may have physiologically adapted to tolerate increased acidity; additionally, high concentrations of organic material in some systems may help to bind toxic aluminum ions that are released under acidic conditions (Dangles, Malmqvist & Laudon, 2004b). Thresholds of acidification effects vary greatly, both between and within taxa: larval midges (Chironomidae: Diptera) have been found to be tolerant to conditions with pH <3 (*e.g.*, Orendt, 1999), while other taxa exhibit striking differences in local adaptation to acidic conditions (Petrin, Laudon & Malmqvist, 2007). The vast majority of pH-related studies have been conducted in the temperate zone, and little is known about acidification tolerance in tropical stream communities (but see Cranston et al., 1997; Roque et al., 2010).

Drought-driven stream pH declines are geographically widespread, occurring in Europe, North America, and Central America, and are predicted to increase with shifting precipitation regimes due to climate change (Laudon et al., 2004; Nordstrom, 2009; Small et al., 2012). These pH declines may be driven by dilution, organic acidity, oxidation of sulfides in soil and rock (Laudon et al., 2004; Nordstrom, 2009), pyrite oxidation (Sammut et al., 1995) and, particularly in the tropics, by biogenic CO_2_ inputs from soil respiration following dry periods (Small et al., 2012) and potentially iron oxidation from lateritic soils (Ganong, 2015). Effects of episodic acidification on stream macroinvertebrates have been described mainly in the context of acidification events driven by anthropogenic acid deposition (*e.g.*, Kowalik et al., 2007) or mining (*e.g.*, MacCausland & McTammany, 2007). Stream macroinvertebrate tolerance to natural drought-driven pH declines remains virtually unstudied save for a CO_2_-induced experimental stream acidification in Puerto Rico, which showed decreased macroinvertebrate abundance and richness at a pH of 5.4 (Klem & Gutiérrez-Fonseca, 2017).

While macroinvertebrates might be expected to be more tolerant to natural pH declines that recur over time than to acute, anthropogenically-driven pH declines, natural drought-driven pH declines are likely to intensify in many areas due to climate change. The Neotropics have been labeled a “hot spot” for climate change (Giorgi, 2006), and drought-driven pH declines are predicted to become increasingly frequent and severe due to increased precipitation variability and dry conditions in coming decades (Rauscher et al., 2008; Min et al., 2011). In lowland Costa Rica, wet-season pH declines can last for months and are negatively correlated with dry-season rainfall in small (first- to third-order) rainforest streams (Small et al., 2012). Extreme acidification events (pH < 4.5) have been observed after unusually dry seasons (Small et al., 2012).

Our objectives were to: (1) determine thresholds of lethal and sub-lethal effects of acidification on benthic macroinvertebrates from lowland streams at La Selva Biological Station (La Selva) in Costa Rica; and (2) examine these thresholds in the context of pH variability from a 16-year stream chemistry dataset, which includes an extreme pH drop resulting from a prolonged dry period associated with a strong ENSO event (1998). We used laboratory mesocosm experiments to test thresholds of macroinvertebrate response to pH declines in two streams: a poorly-buffered stream that is prone to seasonal pH fluctuation (pH 4.3–6.9; typical of many tropical streams) and a stream that is naturally well-buffered (pH 5.1–6.9) due to inputs of bicarbonate-rich regional groundwater. We tested: (1) thresholds of acidification effects on survival of three focal taxa that included the most abundant benthic macroinvertebrates (mayfly naiads, adult shrimp, and midge larvae); and (2) whether chironomid (midge) larvae are uniformly adapted to historic acidification regimes or whether there is local adaptation (*i.e.,* differential tolerance in terms of growth and survival) to acidification between chironomid larvae from the poorly-buffered *vs.* the well-buffered stream. We hypothesized that macroinvertebrates are adapted to acidification within their natal stream’s characteristic pH range (based on the 16-year pH dataset); previous work has shown that La Selva stream insects are adapted to their natal streams’ phosphorus concentration, another key physicochemical parameter that varies across La Selva streams (Small, Wares & Pringle, 2011), suggesting that stream macroinvertebrates may be adapted to the physicochemistry of their natal stream. We predicted that: (1) macroinvertebrate survival and growth rates over would decrease at, or slightly below, the lowest level in this pH range; and (2) the threshold of growth/survival effects would occur at a higher pH level for macroinvertebrates from the well-buffered stream than for macroinvertebrates from the poorly-buffered stream.

## Materials & Methods

### Study site

**Figure 1:**
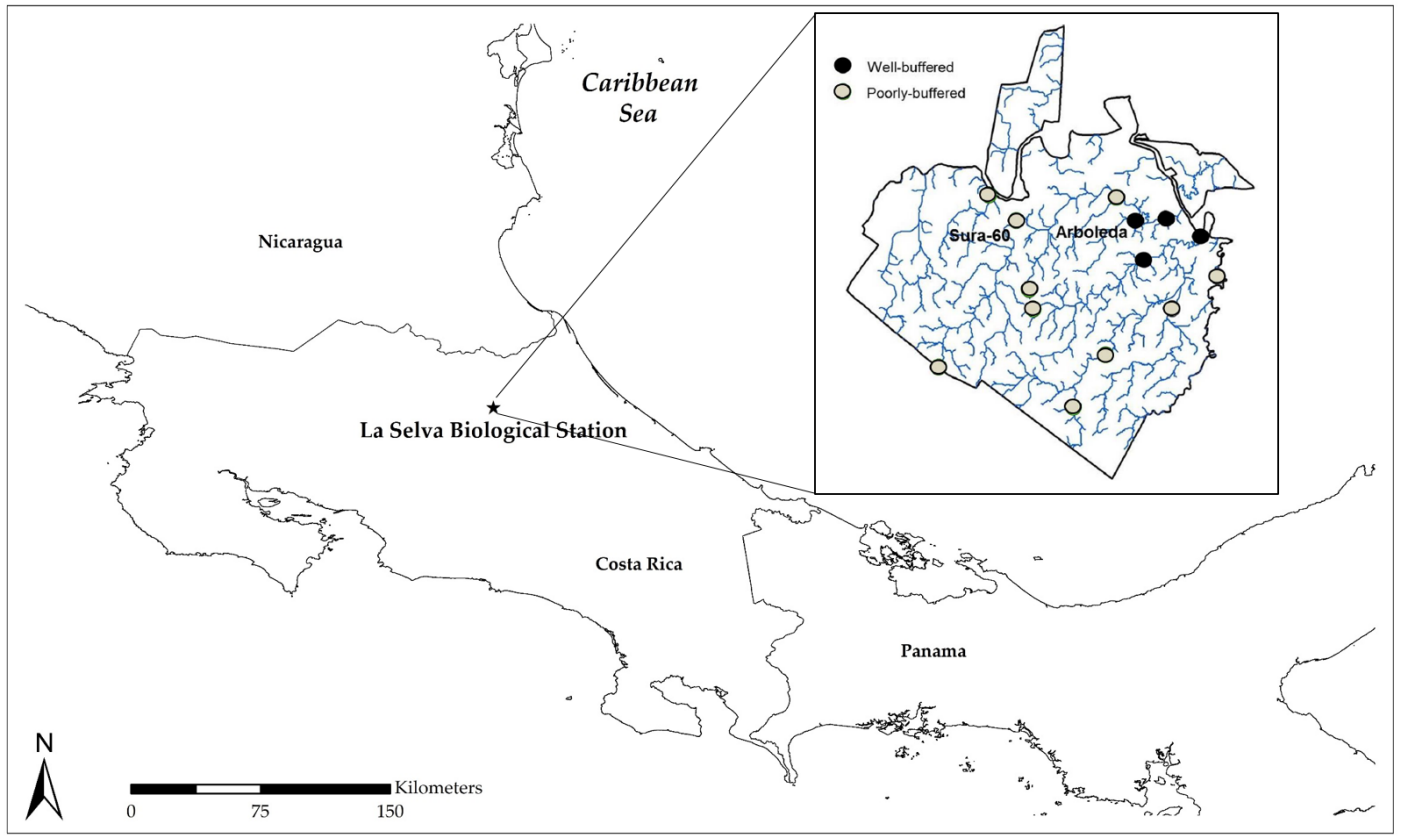
Map of Costa Rica showing the location of La Selva Biological Station (1,536 ha) on Costa Rica’s Caribbean slope; inset shows La Selva stream sites from which longterm pH data have been collected. The poorly-buffered Sura-60 and well-buffered Arboleda are the two sites from which macroinvertebrates were collected for mesocosm experiments.

Our study site was La Selva Biological Station, a 1,536-ha reserve located in lowland rainforest on the Caribbean slope of Costa Rica (Fig. 1A). La Selva is drained by numerous small streams, some of which receive natural interbasin flows of geothermally-modified regional groundwater that emerges in seeps at the base of Pleistocene lava flows (Pringle & Triska, 1991; Pringle et al., 1993). Due to incorporation of ions derived from weathering bedrock, this regional groundwater has high concentrations of many solutes, including bicarbonate, and is naturally well-buffered. In contrast, local groundwater—derived from precipitation within the watershed—is solute-poor and poorly-buffered. Consequently, streams draining La Selva can be solute-poor or solute-rich, depending on whether they receive regional bicarbonate-rich groundwater (Fig. 1B), resulting in very different pH regimes due to the natural buffering capacity of the solute-rich groundwater: well-buffered streams have a relatively high pH (∼6), while poorly-buffered streams have a lower pH that is more prone to fluctuation. Monthly pH measurements at 14 sites have been taken by the same technician since 1997 (methodological details below). Stream pH varies on multiple time scales and is driven by rainfall, with declines due in part to inputs of CO_2_ derived from soil respiration (Small et al., 2012). Transient pH declines of 0.3 to 0.5 units occur immediately after individual precipitation events (Small et al., 2012; Ardón et al., 2013). Longer pH declines occur during the wet season, and low dry-season precipitation is correlated with strong subsequent wet-season stream pH declines that may last for months, with more pronounced pH declines in poorly-buffered streams (Small et al., 2012). Extreme climatic events cause large inter-annual variation: after a severe ENSO-driven drought in 1998, pH in some poorly-buffered streams dropped below 4 (Ramírez, Pringle & Douglas, 2006; Small et al., 2012). Using a one-year monthly dataset from six streams, Ramírez, Pringle & Douglas (2006) showed that insect biomass and density in these streams increased with pH, declining during the major pH drop in 1998. An examination of a 15-year dataset from two poorly-buffered streams indicated that macroinvertebrate richness and abundance were most strongly influenced by stream discharge (Gutiérrez-Fonseca, Ramírez & Pringle, 2018).

Our two study streams were the poorly-buffered Sura-60 and the well-buffered Arboleda, which are similar in terms of size and discharge (Sura-60 0.19 m^3^/s, Arboleda 0.17 m^3^/s; Small & Pringle, 2010). Sura-60 substrate consists mainly of boulders and gravel, while the Arboleda’s substrate is principally sediment with some boulders. These streams have served as focal sites for previous comparative work on aquatic macroinvertebrates (*e.g.*, Small & Pringle, 2010; Small, Wares & Pringle, 2011).

### Stream pH data collection

Monthly pH measurements were taken with a handheld pH meter (Hanna Instruments 9025) at 14 stream sites, including our two focal sites, from April 1997 to December 2013. Ten of these stream sites are classified as poorly-buffered (Fig. 1). Data have been collected by the same field technician since 1997. For the purposes of this paper, seasonal and annual data are considered to begin in January 1998 since data are lacking for the majority of the 1997 dry season.

### Focal taxa

For mesocosm experiments, we selected three focal taxa based upon abundance and respective roles in ecosystem function.

(1) Mayfly naiads (Ephemeroptera: Leptophlebiidae: *Traverella holzenthali*) are common grazers/scrapers in La Selva streams.
(2) Adult shrimp (Decapoda: Palaemonidae: *Macrobrachium olfersii*) are detritivorous macroconsumers that reduce sediment accrual by bioturbation and exert top-down control on algal and insect densities (Pringle & Hamazaki, 1998) as well as leaf decomposition (Rosemond et al., 2001).
(3) Larval midges (Diptera: Chironomidae) are detritivores that constitute 51–80% of invertebrate biomass in stream leafpacks at La Selva (Rosemond et al., 2001). Previous mtDNA analysis demonstrated that the chironomid assemblages in Sura-60 and Arboleda were similar, with 85% of individuals belonging to the same four species (Small, Wares & Pringle, 2011).

### Mesocosm experiments

Macroinvertebrates were collected in April–July 2013 from the poorly-buffered Sura-60 for mesocosm experiments that were conducted in the La Selva laboratory. Treatments included pH 2.5, 3.0, 3.5, 4.0, 5.0, and ambient (∼6.0) for all focal taxa. Additional replicates for Sura-60 chironomids were conducted at pH 3.25 and 3.75. For each experimental replicate, streamwater (1 L) and surface sediment (150 g) collected from Sura-60 were acidified to the target pH *via* addition of 0.3M HCl. Dissolved oxygen and pH were monitored, and pH was adjusted to the target level, as needed, every 8 hrs for the duration of the experiment. Survival was determined for all taxa, and growth was calculated for chironomid larvae using length-mass regressions.

Mayflies (*Traverella holzenthali*) were collected from instream rocks and measured to the nearest tenth of a millimeter using a 1-mm^2^ graph paper grid placed under a dissecting microscope. Initial acidification was achieved using the streamwater-sediment combination described above. Water overlying the sediment was then siphoned into a separate container to avoid clogging mayflies’ gills with continually stirred-up sediment. Each mayfly was placed in its own container of acidified streamwater that included a rock from the stream (to provide biofilm for food) and an aerator attached to an aquarium pump. The streamwater and rock were taken from the mayfly’s natal stream. The experiment (*N* = 3–8 individuals per treatment) lasted 7 days; survival was monitored daily, and final length of all survivors was recorded.

Shrimp (*Macrobrachium olfersii*) were collected using minnow traps baited with cat food and raw chicken wings. Gravid females were released and not used in experiments. Each shrimp was placed in an individual container of acidified streamwater and sediment with an aerator, and survival was monitored daily for seven days (*N* = 4–5 individuals per treatment).

Chironomid analyses were performed according to Small, Wares & Pringle (2011). Larval chironomids were collected from *Ficus insipida* leafpacks incubated instream for 10–13 days. Larvae were measured to the nearest tenth of a millimeter as described above, grouped into 1-mm size classes (ranging from 1 to six mm), and placed in 8 ×10 cm mesh chambers with 90-µm mesh (6 larvae per container, *N* = 5–10 containers per treatment) along with 12 *Ficus* leaf disks conditioned for 5 days in Sura-60. Each container was placed in a separate container of acidified streamwater and sediment for 48 h; survival and length of survivors were recorded, and length-mass regressions were used to calculate percent biomass increase for each container based on mean initial and final larval lengths.

A second experiment was also conducted using chironomid larvae collected from the well-buffered Arboleda focal stream. Larvae were fed *Ficus* leaf disks conditioned for 5 days in the Arboleda, and the experiment was conducted using Sura-60 streamwater and sediment as previously described. Sura-60 streamwater was used since its pH is more amenable to adjustment than water from the well-buffered Arboleda. The control (ambient pH) treatment accounted for any non-pH effects on changes in water chemistry (*e.g.*, variations in phosphorus concentration) on Arboleda chironomid larvae.

### Statistical analysis

To explicitly test the effects of chironomid larval size on survival, we used a general linear mixed-effects model with a logit link to estimate 48-hour survival of individual larvae tested in groups of six larvae per replicate for eight pH treatments (5–10 replicates per treatment). Independent variables were pH treatment, stream of origin (Sura-60 or Arboleda), and mean initial size of larvae in the replicate. We included a random effect on the model intercept for replicate to account for additional, unexplained variation in survival among groups of larvae held in the same container. Initial larval size was scaled by subtracting the mean and dividing by the standard deviation across all replicates. We used a Markov chain Monte Carlo analysis, implemented in JAGS (http://mcmc-jags.sourceforge.net) and R (http://www.r-project.org) software, to estimate model parameters and survival at each pH level. The model was fit using uninformative priors, 3 parallel chains, 50,000 iterations, a 25,000 iteration burn-in, and retaining every third value. Convergence was assessed using the diagnostic statistic “R-hat” (Gelman & Rubin, 1992). We calculated Bayesian 95% credible intervals (analogous to 95% confidence intervals) to assess the precision of survival estimates for each pH level and stream of origin. Model code and priors are included in the supplemental material.

Kruskal-Wallis tests followed by pairwise comparisons were used to compare mayfly and shrimp survival between pH treatments. Mann–Whitney U tests were used to compare survival and initial size of Sura-60 *vs.* Arboleda chironomid larvae for each pH treatment. Multiple linear regressions were used to determine the effects of pH treatment and initial size on growth rate (% biomass increase) for larvae from each site. Post hoc power analyses were conducted for each taxon at each site.

## Results

**Figure 2:**
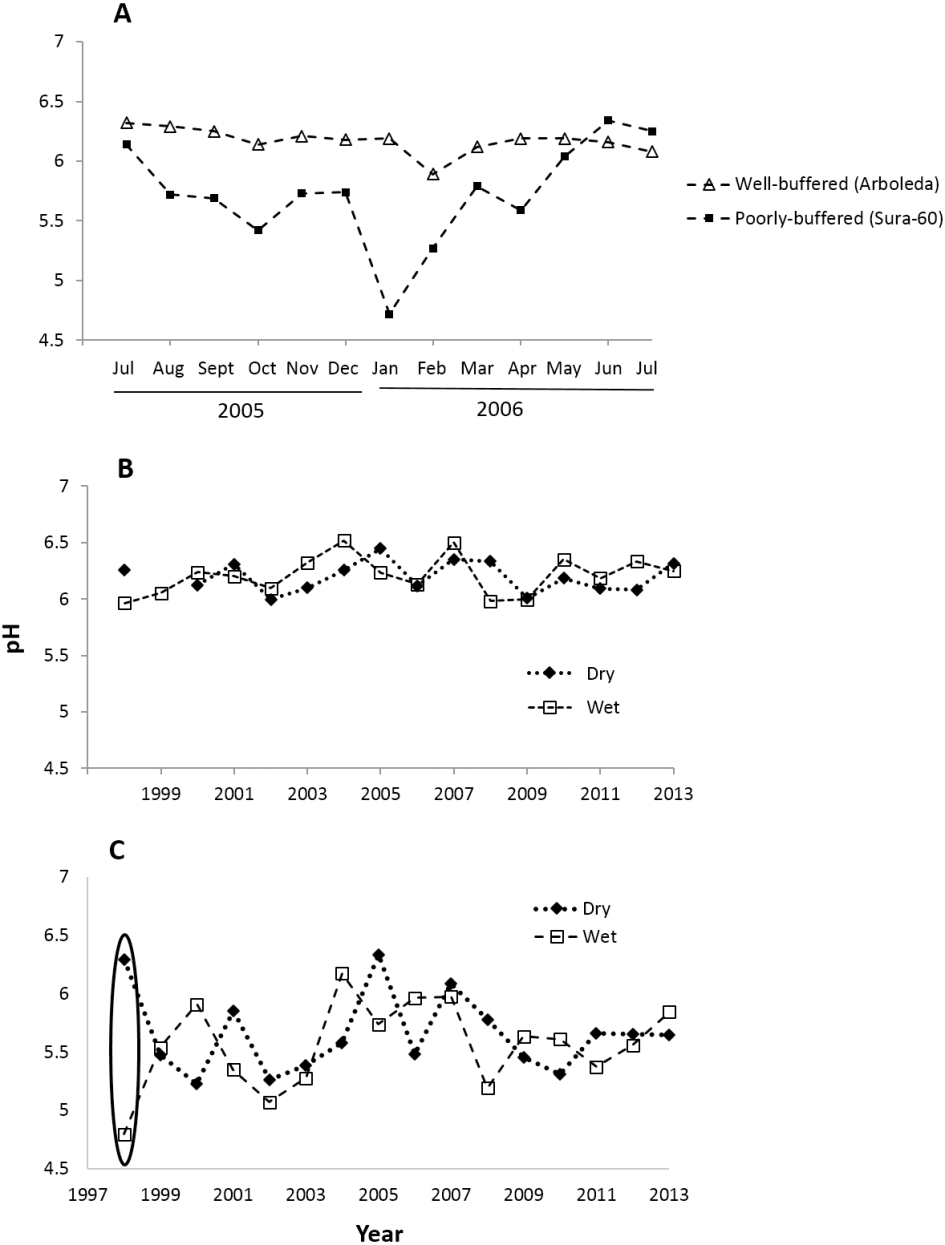
Time series of pH shifts in La Selva streams at multiple time scales. pH shifts occurred in (A) the well-buffered (Arboleda) and poorly-buffered (Sura-60) focal streams at a monthly scale (July 2005–July 2006), (B) the Arboleda at interannual and seasonal timescales (dry (Jan–May) and wet (June–December) season, 1998–2013), and (C) the Sura-60 at interannual and seasonal timescales, with a circle showing the sharp pH shift from dry to wet season after the 1998 El Niño Southern Oscillation Event.

Monthly pH readings from 1997–2013 show a mean pH of 5.63 (range 4.32–6.94) in the poorly-buffered focal stream (Sura-60) and a mean pH of 6.20 (range 5.11–6.92) in the well-buffered focal stream (Arboleda). A yearlong monthly time series (July 2005–July 2006) demonstrates the seasonal variability in pH in both streams: pH in Sura-60 had a range of 1.62 units (pH 4.72 to 6.34) and fluctuated up to 1.02 units in consecutive months, while pH in the Arboleda had a range of only 0.43 unit (pH 5.89–6.32), varying at most 0.30 unit between consecutive months (Fig. 2A).

Fluctuations in pH that we observed at monthly intervals are also evident at seasonal and interannual timescales. In the 16-year time series (1998-2013), the mean annual pH of the well-buffered stream remained relatively constant (range 6.00 to 6.41), with mean monthly pH shifting by 0.01 to 0.35 units between consecutive dry and wet seasons (Fig. 2B). In contrast, mean annual pH in the poorly-buffered stream varied by 1.21 units (range 5.15 to 6.36), and mean monthly pH shifted by 0.06 to 0.68 unit between consecutive dry and wet seasons. The exception was during the 1998 ENSO-driven drought, when mean monthly pH of the poorly-buffered stream during the dry season (January–May) was 6.29, but dropped sharply to 4.80—a decline of 1.49 units—in the ensuing wet season (August–December) (Fig. 2C).

**Figure 3:**
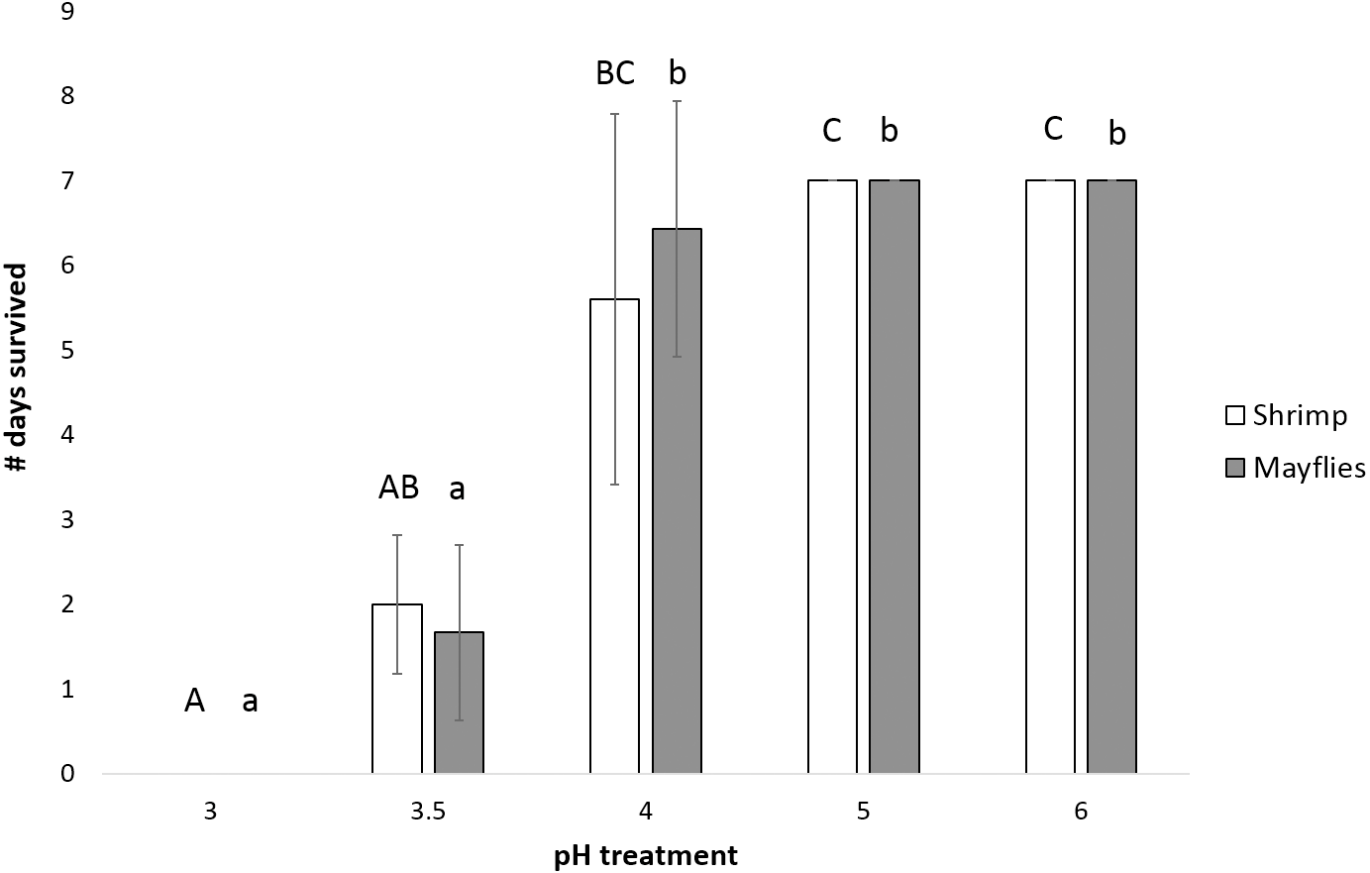
Number of days (mean ± SD) survived by mayflies (Leptophlebiidae: *Traverella holzenthali*) and shrimp (*Macrobrachium olfersii*) from the poorly-buffered Sura-60 under laboratory mesocosm pH regimes. Survival time differed significantly with pH for both taxa (*P* < 0. 001). Mayfly survival time decreased significantly below pH 4 (lower-case letters indicate significant differences between treatments). Shrimp survival time was highest in pH 5 and 6 treatments and significantly lower in the pH 3.5 and 3.0 treatments (capital letters indicate significant differences between treatments).

Mayfly survival time decreased significantly as pH declined (*P* < 0.0001, power = 0.99, effect size = 1.06), with survival significantly longer at and above pH 4 than at pH 3.5 and 3.0 (Fig. 3). Shrimp survival time also decreased significantly with declining pH (*P* = 0.001, power = 0.87, effect size = 1.06), with survival in the pH 4 treatment intermediate between survival at pH 5 and 6 *vs.* pH 3 and 3.5 (Fig. 3).

**Figure 4:**
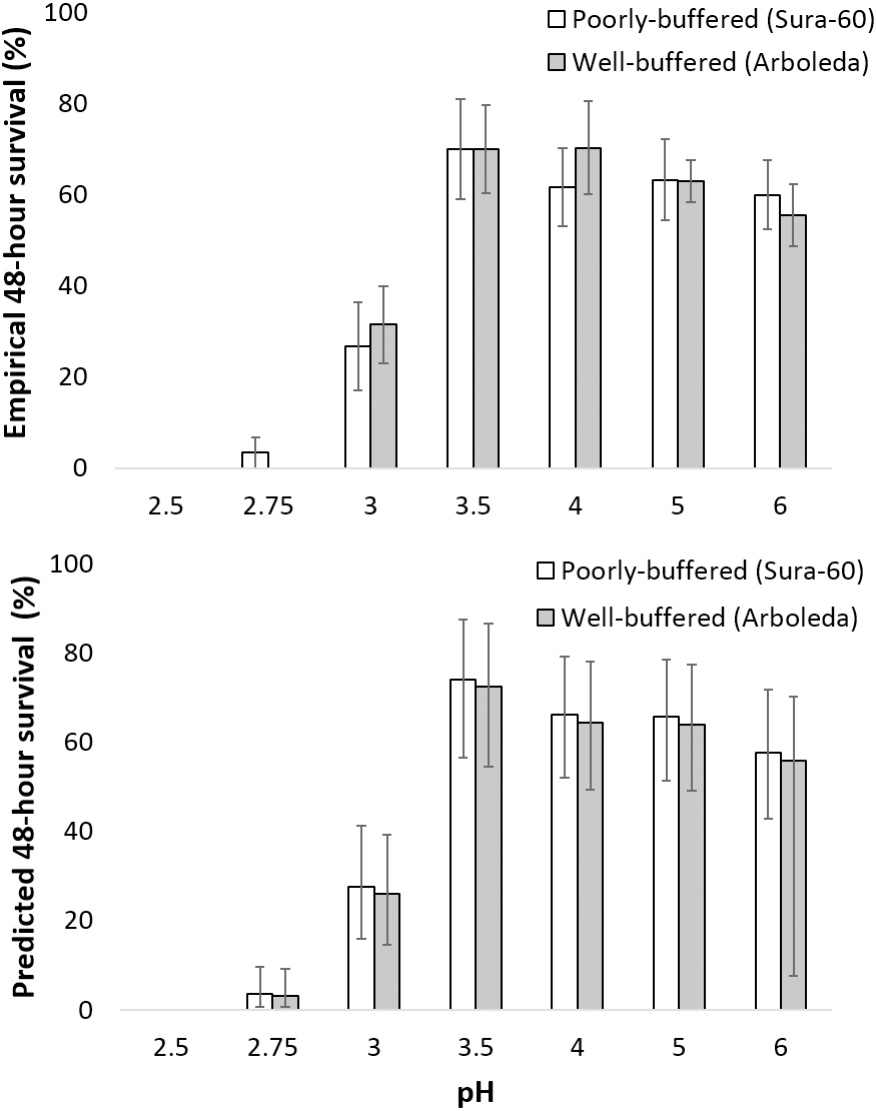
Impacts of pH on chironomid survival. (A) Laboratory survival of larval chironomids (mean % survival over 48 h ±SE) from the poorly-buffered and well-buffered focal streams under different pH regimes. (B) Predicted survival of larval chironomids of average size from the focal streams based on a general linear mixed-effects model; error bars are Bayesian 95% credible intervals (analogous to confidence intervals).

Survival of larval chironomids from both Sura-60 and Arboleda ranged from 56–70% from pH 3.5 to 6.0; survival decreased sharply between pH 3.5 and 3.0, with total mortality at pH 2.5 (Fig. 4A). There was no significant difference in survival, or in initial size, between Sura-60 and Arboleda chironomids in any pH treatment (*P* > 0.05, Sura: power = 0.94, effect size = 0.81; Arboleda: power = 0.96, effect size = 0.90) (Fig. 4A).

Our model of predicted larval chironomid survival yielded similar results: we excluded pH 2.5 due to complete mortality, and 95% credible intervals for pH 2.75 were lower than those for pH 3.0, which in turn were lower than those for pH 3.5 to 6.0; the 95% CI for pH 3.5–6.0 all overlapped (Fig. 4B). The 95% credible interval for stream effect (−0.618 to 0.455) overlapped with 0, indicating no significant stream effect (Table 1). The size effect was positive (95% CI [0.161–0.703]), showing a positive effect of initial chironomid size on survival, and the random effect ranged from [0.275–1.478], indicating a fairly strong “bucket” effect (*i.e.,* unexplained variation among replicates) (Table 1). The effect on survival (exponentiation of the regression coefficient) was less than 1 for pH 2.75 and 3.0, indicating reduced survival, and greater than 1 for all other pH treatments (Table 1). R-hat values for all effect sizes ranged from 1.000 to 1.003, indicating convergence.

**Table 1:** Parameter estimates and 95% credible intervals of a general linear mixed-effect predictive model of larval chironomid survival at different pH levels. pH effect on survival (exponentiation of the regression coefficient) is relative to the intercept.

**Parameter**	**Regression coefficient**	**95% CI**	**Effect on survival**
Stream	−0.08	−0.618 to 0.455	0.923
Initial chironomid size	0.425	0.161 to 0.703	1.530
Random effect	0.763	0.275 to 1.478	2.144
Intercept	0.134	−0.971 to 1.270	–
pH 2.75	−3.680	−5.403 to −2.060	0.025
pH 3.0	−1.126	−2.390 to 0.079	0.324
pH 3.5	0.961	−0.356 to 2.252	2.614
pH 4.0	0.561	−0.686 to 1.762	1.752
pH 5.0	0.537	−0.697 to 1.749	1.711
pH 6.0	0.184	−1.036 to 1.383	1.202

**Figure 5:**
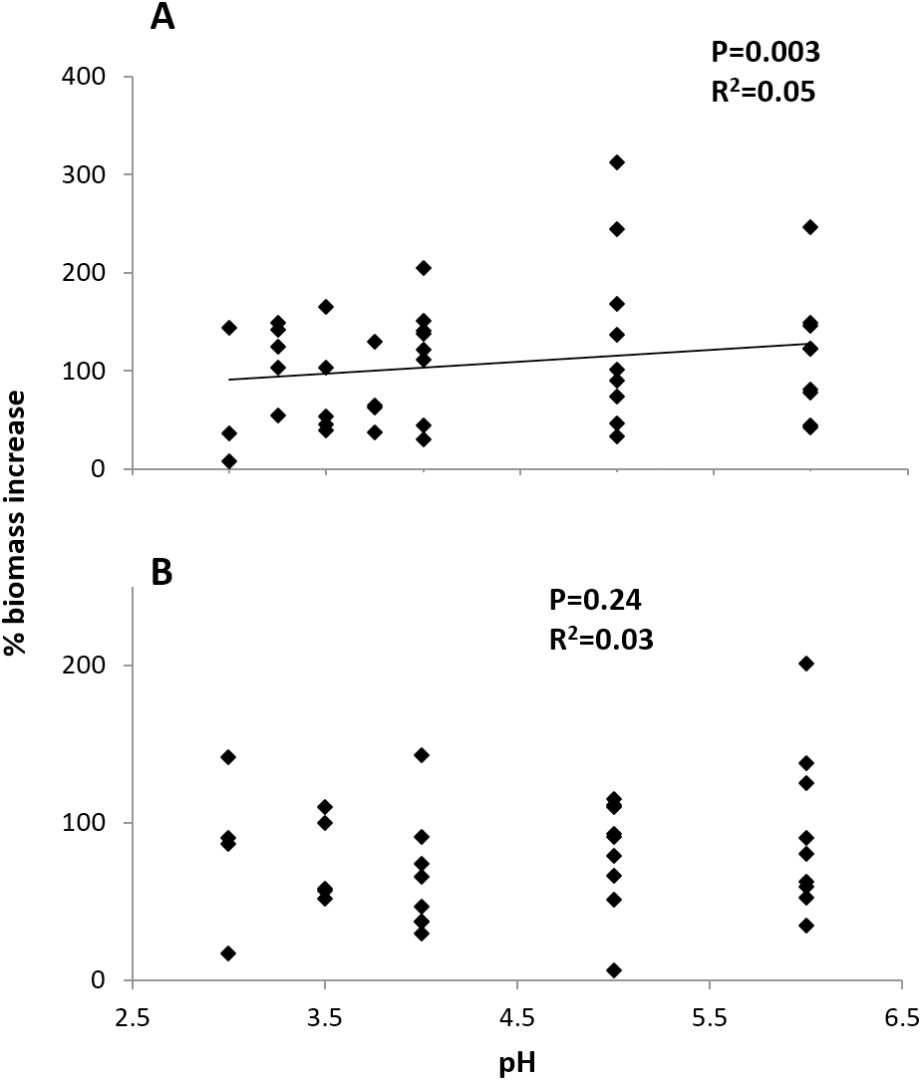
Growth rates (% biomass increase) of larval chironomids in different pH treatments. There was no strong correlation of pH and growth rate in chironomids from (A) the poorly-buffered Sura-60 or (B) the well-buffered Arboleda during 48-hour laboratory mesocosm trials. *P*-values are derived from multiple linear regressions with initial size as a covariate.

Initial size and pH treatment together significantly influenced percent biomass increase at the Arboleda (*P* = 0.04, *R*^2^ = 0.19) and Sura-60 (*P* < 0.001, *R*^2^ = 0.54). In the Arboleda, this trend was driven by the strongly significant negative relationship between growth rate and initial size of larval chironomids (*P* = 0.02, *β* = −0.398), with a nonsignificant effect of pH treatment (*P* = 0.24, *β* = 0.192). In Sura-60, growth rate was also negatively impacted by initial size (*P* < 0.001, *β* = −0.705) but was also positively related to pH (*P* = 0.003, *β* = 0.342) (Fig. 5). Initial size and pH treatment were not significantly correlated at either site.

## Discussion

**Figure 6:**
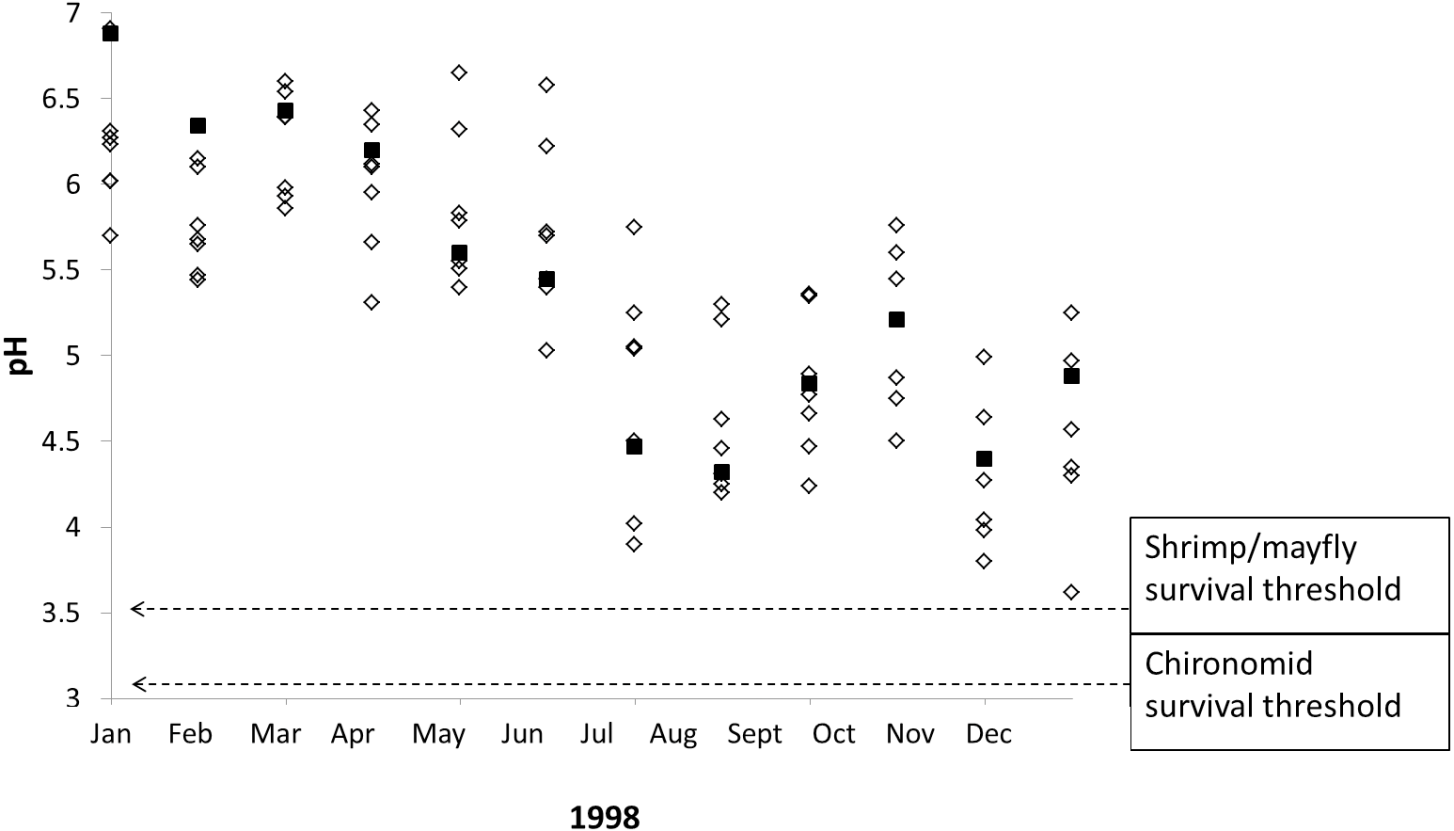
Macroinvertebrate survival thresholds compared to monthly pH readings in La Selva streams during a severe El Niño Southern Oscillation event. The 1998 ENSO event led to episodic acidification in eight poorly-buffered streams (black squares represent Sura-60), causing pH declines that nearly overlap with experimentally determined macroinvertebrate survival thresholds.

Our results indicate that stream macroinvertebrates are tolerant of typical seasonal and interannual pH shifts in poorly-buffered streams, but our data also suggest that stream macroinvertebrates are vulnerable to extreme pH declines caused by severe drought. Monthly pH readings taken from poorly-buffered streams during/after a severe ENSO event in 1998 demonstrate the lowest pH values ever recorded in several streams at La Selva, and these pH values include four instances in which survival thresholds of focal taxa overlap with pH measurements from three streams (Fig. 6), including Sura-100 (pH 3.90) in July, Saltito-100 (pH 3.80) and Sura-100 (pH 3.98) in November, and Carapa (pH 3.62) in December.

Ours is the first study (of which we are aware) to determine thresholds of pH tolerance for macroinvertebrates in streams experiencing extreme natural drought-driven acidification. Severe dry seasons in lowland Costa Rica lead to sharp stream pH declines in subsequent wet seasons (Small et al., 2012): in 1998, intra-annual pH variation (*i.e.,* the difference between the lowest and highest monthly pH) ranged from 1.73 (pH 5.93 to 4.20, at the Taconazo) to 3.01 units (pH 6.91 to 3.90 at Sura-100). Such pH declines are not necessarily restricted to our study system and could be widespread in poorly-buffered streams draining weathered, nutrient-poor tropical soils in areas with seasonal precipitation regimes. These characteristics apply to large areas of the tropics that lack longterm pH records; however, sharp pH declines (from 7.4 to 4) have been recorded in small streams in the Ecuadorean Amazon during floods (Capps et al., 2011).

A comparison of pH declines in La Selva streams and other naturally- and anthropogenically-acidified streams indicates that the magnitude of pH declines (up to 3.01 units) during the extreme acidification event in 1998 is similar to natural pH declines recorded in two other systems, the Ecuadorean Amazon and western US (Table 2). However, the duration of the pH declines in unbuffered streams of La Selva was much longer, with pH values <5 recorded for up to seven months, in one case including pH values <4 for two consecutive months. Notably, the magnitude of natural pH drops driven by precipitation regime (droughts, rainfall, floods, snowmelt) is similar to—and sometimes greater than—the magnitude of declines driven by episodic anthropogenic acidification (Table 2).

Natural drought-driven stream acidification is not only a tropical phenomenon: it also occurs in North American and European streams due to oxidation of sulfide soils in desiccating wetlands (Laudon & Bishop, 2002; Eimers et al., 2007) or acid rock (Verplanck, McCleskey & Nordstrom, 2006; Nordstrom, 2009) (Table 2), as well as in Australian lakes and wetlands (Sammut et al., 1995; Sommer & Horwitz, 2001). These systems are also vulnerable to greater drought-driven pH declines due to climate change (Nordstrom, 2009; Erlandsson, Laudon & Fölster, 2010), so knowledge of the thresholds of macroinvertebrate pH tolerance could have important management implications for temperate as well as tropical streams.

Contrary to our hypothesis, chironomid larvae appear to exhibit general, rather than local, adaptation to stream pH in terms of survival and growth: survival did not differ between sites (Fig. 4), and while pH did significantly impact growth rate at Sura-60, pH explained a very low percentage of variability in growth rate (Fig. 5). Our laboratory results indicating general tolerance of chironomids are supported by two previous field studies involving multiple taxa. First, macroinvertebrate drift (and shrimp movement downstream) during an in-stream acidification experiment at La Selva increased when pH decreased to 4.3–4.35 (Ardón et al., 2013), just above our recorded threshold values. The drifting/downstream-movement response suggests that, in cases of extreme (natural) pH declines, well-buffered downstream reaches could potentially serve as refugia at a landscape scale for organisms moving downstream from poorly-buffered sites (Fig. 1). Second, macroinvertebrate community composition does not differ between well-buffered and poorly-buffered streams (Ramírez, Pringle & Douglas, 2006), suggesting general macroinvertebrate tolerance to low pH. This similarity in macroinvertebrate assemblage composition between well- and poorly-buffered streams contrasts with the findings from other studies in which community composition varied greatly between streams in close proximity with different pH regimes (Lepori, Barbieri & Ormerod, 2003; Kowalik et al., 2007).

The magnitude of natural pH declines within La Selva streams is similar to or greater than that of pH drops in other systems (Table 2), but the ecological impacts—or lack thereof—are more likely due to the source and historical duration of the declines, rather than the magnitude. The prevalence of tolerant taxa in La Selva streams—which have presumably undergone natural acidification for millennia—highlights the importance of historical pH regimes in structuring macroinvertebrate communities (Collier et al., 1990; Dangles et al., 2004a; Dangles, Malmqvist & Laudon, 2004b). Comparison of our findings with pH tolerances from other systems (Table 3) shows that systems that have experienced natural acidification for millennia, such as mountain streams with pH declines driven by snowmelt, often include macroinvertebrate communities that are highly resistant to acidification (Lepori, Barbieri & Ormerod, 2003; Petrin, Laudon & Malmqvist, 2007). In contrast, macroinvertebrates in streams that have historically exhibited circumneutral pH levels are often severely impacted by sudden exposure to acidic conditions caused by acid rain or acid mine drainage (Guérold et al., 2000; Kowalik & Ormerod, 2006; MacCausland & McTammany, 2007).

**Table 2:** Examples of streamflow-driven episodic pH declines in streams (data from La Selva in bold). Studies are classified based on episodic declines driven primarily by natural (*i.e.*, non-aciddeposition) causes or mainly by acid deposition.

**Type**	**Cause**	**Magnitude**	**Duration**	**Site**	**Study**
Natural	Floods	3.4 units (7.4 to 4)	Hours	Ecuador	Capps et al. (2011)
	***Drought/rain: CO***_***2***_ ***, other***	***1.73 units (5.93 to 4.20)*** ***to 3.01 units (6.91 to 3.90)***	***Months***	***Costa Rica***	Small et al. (2012)
	Drought/rain: acid rock oxidation	3 units (7.8 to 4.8)	Hours	Nevada (USA)	Nordstrom (2009)
	Drought/rain: DOC inputs, sulfide oxidation in wetlands (also minor S deposition)	1.0–2.4 units (pH ≥ 4.25)	N/A	Sweden	Laudon & Bishop (2002)
	Drought/rain: dilution, acid sulfate soil runoff (post-drought); minor NO_3_^−^, DOC, and sea salt inputs	0.4–0.9 unit (pH ≥ 4.5)	N/A	Sweden	Erlandsson, Laudon & Fölster (2010)
	Snowmelt: dilution, some NO_3_^−^ input	Up to 1.4 units (6.7 to 5.3)	N/A	Switzerland	Lepori, Barbieri & Ormerod (2003)
	Storms: sea salt input	0.5 unit (4.90–4.45)	Months	Norway	Hindar et al. (1995)
Anthropogenic	AMD	Up to 1.99 units (6.93 to 4.94)	N/A	Pennsylvania (USA)	MacCausland & McTammany (2007)
	Drought/rain, acid deposition	Up to 1.5 units (6.7 to 5.2)	Hours	Scotland	Kowalik et al. (2007)
	Rain, acid deposition	Up to 1.63 units (6.20 to 4.57)	N/A	Northeastern USA	Baker et al. (1996)
	Drought/rain, acid deposition	1.1 units (5.86 to 4.78)	Hours/days	New York (USA)	Baldigo et al. (2009)

**Notes.**

AMD, acid mine drainage.

**Table 3:** Survival of Chironomidae (examples), Ephemeroptera (examples), and Palaemonidae/Atyidae (all known studies) under acidic conditions.

**Taxon**	**Subfamily/ genus/species**	**Acid source and stream location**	**pH survived**	**Experiment or observation**	**Study**
Chironomidae	Chironominae, Tanytarsinae, Orthocladiinae, Tanypodinae	AMD, Australia	2.9	observation	Cranston et al. (1997)
	Chironominae, Tanytarsinae, Orthocladiinae, Tanypodinae	acid precipitation, Canada	<4.5	observation	Mackay & Kersey (1985)
	*Limnophyes*	natural and acid precipitation	2.79	observation	Orendt (1999)
	*Corynoneura, Tanytarsus*	natural and acid precipitation	∼3	observation	Orendt (1999)
	*Naonella, Eukiefferiella*	naturally acidic and AMD, New Zealand	3.4	observation	Winterbourn & McDiffett (1996)
	*Chironomus riparius*	naturally-acidic tundra ponds, Canada	2.8 (20% mortality in 48 h, ∼60% after 400 h)	experiment	Havas & Hutchinson (1982)
Ephemeroptera: Leptophlebiidae	*Leptophlebia*	acid precipitation, Canada	4.3–4.5	observation	Mackay & Kersey (1985)
	*Deleatidium*	naturally acidic and AMD, New Zealand	3.4	observation	Winterbourn & McDiffett (1996)
	*Deleatidium*	AMD, New Zealand	3.5–4.0	experiment	O’Halloran, Cavanagh & Harding (2008)
	*Choroterpes picteti*	AMD, Portugal	4.8 (48-h LC_50_)	experiment	Gerhardt, Janssens de Bisthoven & Soares (2005)
Shrimp: Palaemonidae, Atyidae	*Atyaephrya desmaresti* *Macrobrachium rosenbergii*	AMD, Portugal China	LC_50_ 5.8 LC_50_ 4.0-4.8	experiment experiment	Gerhardt, Janssens de Bisthoven & Soares (2004) Chen & Chen (2003)

**Notes.**

AMD, acid mine drainage.

Increased incidence of severe pH declines due to climate change would likely have ecosystem-level effects due to potential loss of key taxa. The lower tolerance of mayflies and shrimp (Fig. 3) compared to chironomids (Fig. 4) suggests that extreme pH declines might simplify macroinvertebrate communities as mayflies and shrimp die, drift, or move downstream to escape low pH levels. Shrimp can play key roles in reducing sediment accrual and driving insect densities and algal community composition (Pringle & Hamazaki, 1998; Rosemond et al., 2001), as well as contributing up to 94% of stream invertebrate biomass and 40% of macroinvertebrate secondary production (Snyder, 2012). Therefore, the loss of *Macrobrachium* could potentially cause a significant shift in ecosystem structure and function due to changes in algal taxa, higher chironomid density, and greater sediment accrual.

## Conclusion

Our work provides evidence that macroinvertebrates in La Selva streams are tolerant to naturally low pH levels, but are vulnerable to the extreme pH declines (pH < 4) observed following severe droughts. Chironomid growth and survival were unaffected by pH regimes above 3.0, indicating tolerance to pH levels experienced in the poorly-buffered stream during natural seasonal acidification, while shrimp and mayfly survival were impacted at pH levels below 4. Given that droughts are predicted to increase in frequency and severity in the future, climate change potentially threatens macroinvertebrate taxa in these streams. These findings also signal the need for further research in streams worldwide that are vulnerable to drought-driven pH declines.

## Supplemental Information

10.7717/peerj.11955/supp-1 Supplemental Information 1 Chironomid survival model code

10.7717/peerj.11955/supp-2 Supplemental Information 2 Raw data

## Additional Information and Declarations

### Data Availability

The following information was supplied regarding data availability: Data are currently available in the Supplemental File and is available at Zenodo: Ganong, Carissa. (2021). Data for “Thresholds of acidification impacts on macroinvertebrates adapted to seasonally acidified tropical streams” [Data set]. Zenodo. https://doi.org/10.5281/zenodo.5245706.
