# Supplementary material for: Thresholds of acidification impacts on macroinvertebrates adapted to seasonally acidified tropical streams: potential responses to extreme drought-driven pH declines
Source: PeerJ. 2021 Nov 23;9:e11955. doi: 10.7717/peerj.11955 (PMC8621781; doi:10.7717/peerj.11955)
Supplement: Supplemental Information 1 [file peerj-09-11955-s001.docx]

**Chironomid survival model code**

## mixed logistic regression, mortality by ph level, random effect of replicate

datafull<-Data_for_JAGS_8_May_2020 #

dataformary<-subset(datafull,datafull$pH != 2.5) #exclude ph=2.5, b/c none survived

dataformary$pHclass<-1 # for ph=2.75

dataformary$pHclass<-ifelse(dataformary$pH ==3.00,2,dataformary$pHclass)

dataformary$pHclass<-ifelse(dataformary$pH ==3.25,3,dataformary$pHclass)

dataformary$pHclass<-ifelse(dataformary$pH ==3.50,4,dataformary$pHclass)

dataformary$pHclass<-ifelse(dataformary$pH ==3.75,5,dataformary$pHclass)

dataformary$pHclass<-ifelse(dataformary$pH ==4.00,6,dataformary$pHclass)

dataformary$pHclass<-ifelse(dataformary$pH ==5.00,7,dataformary$pHclass)

dataformary$pHclass<-ifelse(dataformary$pH ==6.00,8,dataformary$pHclass)

dataformary$survival<-(1 - dataformary$Survived) ## recode data so that 1=survived

dataformary$size.s<-scale(dataformary$Initial.size..mm.)

dataformary$newrep<-dataformary$Rep-10

dataformary$newrep<-ifelse(dataformary$newrep>60,dataformary$newrep-7,dataformary$newrep)

dataformary$site<-ifelse(dataformary$Stream=="A",2,1) #site is 2 for chiros from well-buffered stream

#Summarize raw data

library(dplyr)

size_by_ph<-dataformary%>%group_by(Stream,pH)%>% summarize(mean_survival=mean(survival), mean_size=mean(Initial.size..mm.))

size_by_ph

# # Groups: pH [8]

# Stream pH mean_survival mean_size

#<chr> <dbl> <dbl> <dbl>

1 A 2.75 0 3.57

2 A 3 0.315 3.81

3 A 3.5 0.7 3.62

4 A 4 0.704 4.12

5 A 5 0.630 3.92

6 A 6 0.556 3.81

7 S 2.75 0.0333 3.75

8 S 3 0.267 3.41

9 S 3.25 0.867 3.12

10 S 3.5 0.7 3.29

11 S 3.75 0.667 3.61

12 S 4 0.617 3.77

13 S 5 0.633 3.47

14 S 6 0.6 3.94

#############################################

y<-dataformary$survival #636 individuals

Rep<-dataformary$newrep #636 values

numRep<-unique(Rep) #106 reps

nrep=length(numRep)

repdata<-dataformary%>%group_by(newrep)%>%summarise(pH=mean(pHclass), size.s=mean(size.s), site=mean(site))

pH<-repdata$pH #106 values

size.s<-repdata$size.s #106 values

site<-repdata$site #106 values

nind<-length(y) #636

library(R2jags)

library(rjags)

sink("chiro.survival.new.jags")

cat("

model {

# Likelihood

for (i in 1:nind){

y[i] ~ dbern(mu1[i]) #observed survival is draw from a probability

mu1[i] <- surv[Rep[i]] #the probability

} #i

# Priors and constraints

for (i in 1:nrep){

logit(surv[i]) <- alpha + beta.pH[pH[i]] + beta.size*size.s[i] + beta.buffered[site[i]] + epsilon[i] ## survival with random effect for rep

} #i

#random effect for rep

for (s in 1:nrep){ #

epsilon[s]~dnorm(0,tau.r)

} #s

sigma.r~dunif(0,10)

tau.r<-pow(sigma.r,-2)

sigma2.r<-pow(sigma.r,2)

# for survival parameters

alpha ~ dnorm(0, 0.37)I(-15, 15) # Priors for intercept; bounded to fall between -15 and 15

beta.size ~ dnorm(0, 0.37)I(-15, 15)

beta.buffered[1]<-0 # Corner constraint

beta.buffered[2] ~ dnorm(0, 0.37)I(-15, 15) # Prior for difference in survival in Arboleda vs Sura

surv.s.2.75 <- 1 / (1+exp(-alpha-beta.pH[1])) # Back-transformed survival for ave size, ph=2.75, and so on, in SURA

surv.s.3.0 <- 1 / (1+exp(-alpha-beta.pH[2]))

surv.s.3.25 <- 1 / (1+exp(-alpha-beta.pH[3]))

surv.s.3.5 <- 1 / (1+exp(-alpha-beta.pH[4]))

surv.s.3.75 <- 1 / (1+exp(-alpha-beta.pH[5]))

surv.s.4.0 <- 1 / (1+exp(-alpha-beta.pH[6]))

surv.s.5.0 <- 1 / (1+exp(-alpha-beta.pH[7]))

surv.s.6.0 <- 1 / (1+exp(-alpha-beta.pH[8]))

surv.a.2.75 <- 1 / (1+exp(-alpha-beta.pH[1]-beta.buffered[2])) # Back-transformed survival for ave size, ph=2.75, and so on, in ARBOLEDA

surv.a.3.0 <- 1 / (1+exp(-alpha-beta.pH[2]-beta.buffered[2]))

surv.a.3.25 <- 1 / (1+exp(-alpha-beta.pH[3]-beta.buffered[2]))

surv.a.3.5 <- 1 / (1+exp(-alpha-beta.pH[4]-beta.buffered[2]))

surv.a.3.75 <- 1 / (1+exp(-alpha-beta.pH[5]-beta.buffered[2]))

surv.a.4.0 <- 1 / (1+exp(-alpha-beta.pH[6]-beta.buffered[2]))

surv.a.5.0 <- 1 / (1+exp(-alpha-beta.pH[7]-beta.buffered[2]))

surv.a.6.0 <- 1 / (1+exp(-alpha-beta.pH[8]-beta.buffered[2]))

beta.pH[1] ~ dnorm(0, 0.37)I(-15, 15)

beta.pH[2] ~ dnorm(0, 0.37)I(-15, 15) # Prior for effect of survival at each ph 3, adds to intercept

beta.pH[3] ~ dnorm(0, 0.37)I(-15, 15)

beta.pH[4] ~ dnorm(0, 0.37)I(-15, 15)

beta.pH[5] ~ dnorm(0, 0.37)I(-15, 15)

beta.pH[6] ~ dnorm(0, 0.37)I(-15, 15)

beta.pH[7] ~ dnorm(0, 0.37)I(-15, 15)

beta.pH[8] ~ dnorm(0, 0.37)I(-15, 15)

}

",fill = TRUE)

sink()

# Bundle data

jags.data <- list(y = y, size.s = size.s, Rep=Rep, pH=pH, nind = nind, nrep=nrep, site=site)

# Initial values

inits <- function(){list(alpha = rnorm(1), beta.size = rnorm(1), beta.buffered = c(NA, rnorm(1)),

beta.pH = c(rnorm(1), rnorm(1), rnorm(1),rnorm(1),rnorm(1),rnorm(1),rnorm(1),rnorm(1)), sigma.r=runif(1,0,5))}

# Parameters monitored

parameters <- c("alpha","beta.pH", "beta.size", "beta.buffered", "surv.s.2.75", "surv.s.3.0",

"surv.s.3.25", "surv.s.3.5", "surv.s.3.75","surv.s.4.0", "surv.s.5.0", "surv.s.6.0",

"surv.a.2.75", "surv.a.3.0",

"surv.a.3.25", "surv.a.3.5", "surv.a.3.75","surv.a.4.0", "surv.a.5.0", "surv.a.6.0","sigma2.r", "sigma.r")

# MCMC settings

ni <- 50000

nt <- 3

nb <- 25000

nc <- 3

# Call JAGS from R

chiro_surv_wtf <- jags(jags.data, inits, parameters, "chiro.survival.new.jags", n.chains = nc, n.thin = nt, n.iter = ni, n.burnin = nb)

# Summarize posteriors

print(chiro_surv_wtf, digits = 3)

#compare to ML fit

modelb<-glmer(survival~(1|Rep)+factor(pH)-1 +size.s+ site, family=binomial, data=dataformary)

summary(modelb) # note here, the "p" values are for the coefficients being different from 0, = survival prob =0.5

modelb@beta # the regression coefficients

odds<-exp(modelb@beta)

print(surv_estimates<-odds/(1+odds))
